# The Significance of Software Engineering to Forecast the Public Health Issues: A Case of Saudi Arabia

**DOI:** 10.3389/fpubh.2022.900075

**Published:** 2022-08-18

**Authors:** Haneen Hassan Al-Ahmadi

**Affiliations:** Software Engineering Department, College of Computer Science and Engineering, University of Jeddah, Jeddah, Saudi Arabia

**Keywords:** public health, artificial neural network, support vector machine, forecasting, Saudi Arabia

## Abstract

In the recent years, public health has become a core issue addressed by researchers. However, because of our limited knowledge, studies mainly focus on the causes of public health issues. On the contrary, this study provides forecasts of public health issues using software engineering techniques and determinants of public health. Our empirical findings show significant impacts of carbon emission and health expenditure on public health. The results confirm that support vector machine (SVM) outperforms the forecasting of public health when compared to multiple linear regression (MLR) and artificial neural network (ANN) technique. The findings are valuable to policymakers in forecasting public health issues and taking preemptive actions to address the relevant health concerns.

## Introduction

Health policymakers are concerned about economic issues and management of the health sector. Alone, health does not mean the lack of illness, but rather the ability of people to be an asset for the nation in terms of intrinsic and instrumental value (1). The health sector comprises of complex technologies that serve public health and wellbeing. The adoption of developmental technologies in a country is expensive and stimulates the health sector finances. An important factor, which causes health issues all over the world and cannot be ignored, is particulate matter 2.5 (PM2.5) in air. For a long time, a causal affiliation has been found among air pollution and health problems in humans. In Europe, the most problematic pollution is particulate matter 2.5 (PM2.5) and ground-level ozone (O_3_). The foremost causes of these toxins are gas and energy outputs used by different industries or population. Air pollution is highly associated with cardiovascular illnesses, strokes (2), and respiratory diseases (3). Furthermore, children are also at risk of neurological development issues from PM2.5 pollutants (4). A study carried out in Australia found that reducing PM2.5 in the environment decreases premature death rates, indicating that better investments and sustainable growth steps should be taken by economies to prevent public health crises (5).

This study incorporates the prediction and analysis of Saudi Arabian healthcare issues and risks for the period of 2008–2019. Our primary aim is to analyze the variables causing health risks, which ultimately lead to health risks for the country. Second, we further elaborate on the importance of each variable on medical expenditures, population, carbon dioxide (CO_2_) emissions, and gross domestic product (GDP). The health sector affects economic development through the health of workers and their consequent economic productivity, the country's financial resources, and also by improving educational opportunities for the youth population, which can be asset to the country (1). We apply three methods, namely, the artificial neural network (ANN), support vector machine (SVM), and multiple linear regression (MLR), to find the best model in forecasting future risks. Last, we present the forecasting of next year's public health issues by using the best forecasting tool, which aids policymakers in their health sector decisions and management of financing.

### Overview of Saudi Arabia

**Figure 1:**
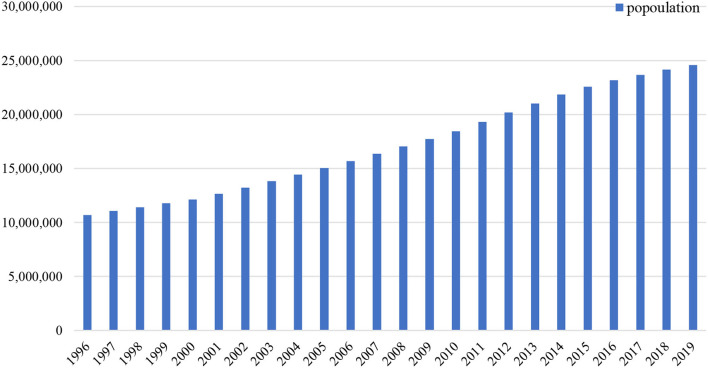
Population rising trend (1996–2019) (Source: world development indicators).

Currently, Saudi Arabia (KSA) has an estimated population of ~32.6 million, making it the largest country among the Gulf Cooperation Council (GCC) countries (World Development Indicators). Saudi Arabia is also believed to be the fastest growing population among the GCC countries and is anticipated to reach 35 million people by 2050. Figure 1 depicts the increasing trend. Almost 60% of the population is aged 35 years or younger, which increases the steady demand of healthcare facilities (6). As per Vision 2030, many fundamental structural reforms have been introduced by the government, which also include the healthcare sector. The healthcare system includes both the private and public, both of which provide good quality services. Moreover, 60% of the sector is publicly owned and handled by the government's Ministry of Health. With the population in KSA having significantly increased between 1980 and 2015 and along with its current protectory, the population size is likely to impact the quality, quantity, and type of healthcare services available. Diseases among the Middle East and North Africa (MENA) region have drastically increased due to lack of health awareness.

**Figure 2:**
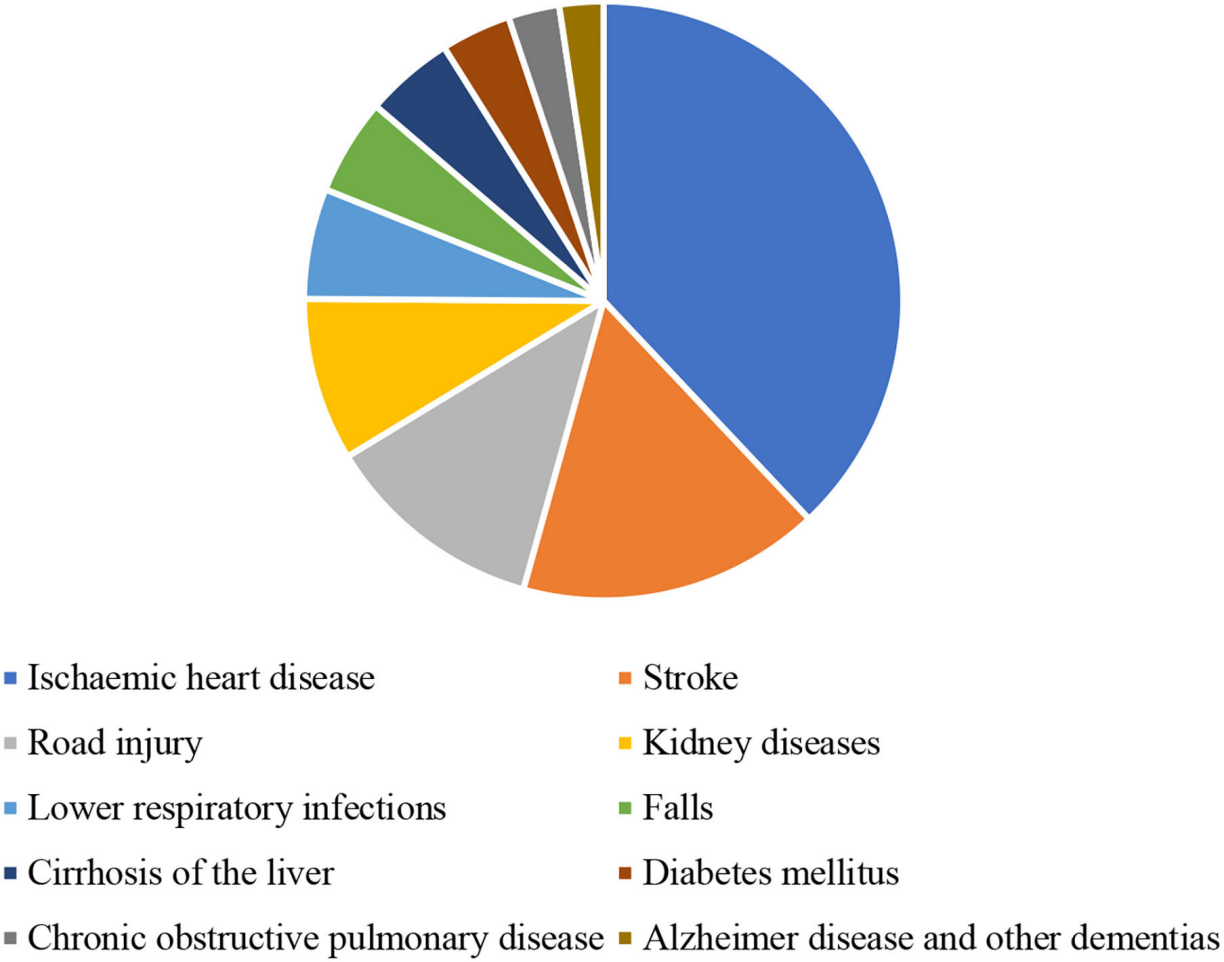
Death rate in Saudi Arabia (per 100,000) in 2019 (Source: world development indicators).

Another reason to study the health sector is that health issues are rising in KSA due to lifestyle diseases such as diabetes, heart strokes, and other non-communicable diseases. As presented by Figure 2, the death rate from these serious illnesses is higher than other diseases. According to statistics, in 2014, over 422 million people were diagnosed with diabetes worldwide and the MENAs had 38.7 million diabetic patients in 2017. Hence, these numbers are expected to rise above 70 million by 2024. Another health-related factor in 2016 is that KSA has an obesity rate of 34.5% among adults, which was again highest in the MENA area. Dealing with the rising health expenditure for the country, especially in private sector which offers several opportunities to investors, operators, and health professionals, is yet another challenge for KSA. High capital costs for attracting high-quality doctors and nurses is important, while maintaining good healthcare services. Early in 2010, Saudi Arabia increased investments in its health sector with a surge of 68% of gross domestic product (GDP) in the national government healthcare expenditure. This was notably greater than those in northern Africa (7).

Currently, Saudi Arabia is delivering free healthcare services to all the citizen and expatriates hired in public sectors, primarily under the Ministry of Health. The government requires some private coverage by employers for the expatriates employed in private organizations. Healthcare services are considered citizen rights and are mostly covered by government expenses. Managing GDP for the country—which ultimately leads to many services provided by government—is another important challenge for the government. Healthcare investment challenges are also associated with rising health issues.

## Literature Review

Numerous studies have been conducted to monitor the public health issues (8–11). As health issues are closely related to good health policies, however, it is important to determine the factors which are affecting the public health (12–14). While focusing on the child health issues, several researchers attempted to investigate the determinants of child health issues (15–17). Similarly, Gilliland et al. (18) examined the impact of air quality on child health, which proposes that improvement in air quality helps to minimize the lungs issues.

In the recent years, emerging air pollutants around the world and their impact on public health are the key area of study (19–22). In few important aspects such as indoor and outdoor air quality, the WHO (23) reports that 3.8 million deaths are caused by higher risk of diseases, which generate through indoor air pollution. Tsakas et al. (24) reported that decreased ventilation rate, modern living, and use of synthetic building material are also cause of worsening indoor air quality. But, it is an issue to be raised as reduction in the urban living is yet another emerging issue. Eisner and Balmes (25) mentioned that air pollution hazard arises from both the natural and human activities source and physicians are needed to know that exposure to these pollutants is rising, especially causing lungs and respiratory issues in upcoming generations. Sarwar et al. (26, 27) documented the nexus among carbon emission and health issues.

In case of Saudi Arabia, several studies are addressing the issues separately and creating awareness among the people for health-related risk. Alquaiz et al. (28) addressed in the study about women health risk associated with non-communicable diseases and found in survey that women are to be at greater risk in coming era than men. Similarly, Subhan et al. (29) concluded that young men are at health risk due to smoking habits. Nafees et al. (30) mentioned that smoker's household environment is three times exposed to hazard air pollutants particles in Saudi Arabia. Al Daajani et al. (31) and Moradi-Lakeh et al. (32) documented the early age health issues in children in Saudi Arabia. Al-Hanawi et al. (33) stated that human resource development is major investment in Saudi Arabian concern, which is due to the arising concerns in health issues. Unhealthy lifestyle, due to lack of awareness, making Saudi population prone toward health risk; however, it is important to find the factors and put more investments in health sector (34, 35).

Walston et al. (36) describe that Saudi population is growing rapidly, which leads to more healthcare expenditures for the country. Population growth is a threat to social and environmental factors (37) and lead to health issues by burden of diseases (38). Chemical pollution, environmental pollution, and water pollution all are causes to the burden of diseases hence, it causes health risk to the population. Mahmood et al. (39) stated that environmental policies needed to be re-evaluated, as Saudi Arabia is an oil generating country that leads more environmental degradation (40). Health issues has direct link to clean environment (20, 26, 27, 41, 42) and many more confirmed this relation. In this study, we used multiple linear regression (MLR) and machine learning tools such as support vector machine (SVM) and artificial neural network (ANN) for forecasting health issues. Table 1 presents the previous studies, which incorporated the machine learning tools for forecasting.

**Table 1:** Overview of support vector machine (SVM) and artificial neural network (ANN) studies.

**References**	**Goal**	**Method**	**Findings**
Vijayarani and Dhayanand (43)	Kidney disease prediction using SVM and ANN algorithms	The interest of this research paper is to forecast kidney diseases by applying Support Vector Machine (SVM) and Artificial Neural Network (ANN).	ANN is better than SVM.
Esmaeily et al. (44)	Comparing three data mining algorithms for identifying the associated risk factors of type 2 diabetes	In this research, artificial neural network (ANN), support vector machines (SVMs), and multiple logistic regression (MLR) models were used, using demographic, anthropometric, and biochemical features.	ANN achieves better results.
Madhuravani et al. (45)	Prediction exploration for coronary heart disease aid of machine learning	The tentative result is on three forecast methods like SVM, K-NN and ANN. It is to generate and recognize the coronary heart disease using three diverse organize machine learning.	Several techniques have been applied for calculation methods, the finest accuracy found in ANN, the greatest precision in K-NN and the top recall in ANN.
Hooda and Mann (46)	Examining the Effectiveness of Machine Learning Algorithms as classifiers for predicting disease severity in data warehouse environments	The Artificial Neural Network (ANN) and Support Vector Machine (SVM) are performed to generate improved input influences (weights and bias) for the choice of best kernel to categorize the data for additional diagnosis.	SVM performed better than ANN
Son et al. (47)	Application of support vector machine for prediction of medication adherence in heart failure patients	They function a Support Vector Machine (SVM), a machine-learning method valuable for data sorting.	SVM modeling is a capable classification method for forecasting medication adherence in heart failure patients
Mello-Román et al. (48)	Predictive models for the medical diagnosis of dengue: a case study in Paraguay	They used Artificial neural networks (ANN) and support vector machines (SVM) as supporting tools for medical diagnosis.	In their results, SVM polynomial attained outcomes above 90% for accuracy, sensitivity, and specificity.

Existing literature focused on SVM and ANN for specific disease, instead of overall public health issues. Either specific health concerns are important, but it lacks to drive the overall health issues of a specific country or region. Without an accurate prediction of health issues, it is nearly impossible to allocate the exact health budget, as well as to take preemptive measures. However, this first contribution of existing study is to fill this gap, in case of Saudi Arabia. Second, this study contributes by using the linear regression and machine learning tools to predict the public health in case of Saudi Arabia, whereas previous literature has not focused on Saudi Arabia.

## Data and Methodology

This study employs multiple linear regression (MLR) and two main machine learning tools that are support vector regression (SVR) and ANN, which aim to forecast the health issues. Although, SVR and ANN are gaining interest among researcher, the least error in prediction makes it fits to be employed in this study. The data set contains the period of January 2008 to October 2019 and the sources of data are given in Table 2.

**Table 2:** Source of data.

**Abbreviation**	**Variable**	**Source**
Health	Exposure to PM2.5	OECD
CO_2_	Production-based CO_2_ intensity, energy-related CO_2_ per capita (Tons)	OECD
GDP	Real GDP	WDI
Health Expenditure	Current health expenditure per capita (current US$)	WDI
Population	Population ages 15–64, total	WDI

### Regression Model

This statistical model is basically a technique to identify the relationship between dependent and independent variables. Regression analysis is mainly used for binary theoretically dissimilar purposes. First, it is used for the predictions, which are nearly similar to machine learning tool. Second, in some circumstances, regression analysis concludes causal relationships among the independent and dependent variables. Linear model in equation (1) is as follows:


(1) $$\begin{array}{r} Health\;Issue=\;\beta_{0}+\beta_{1}{Health\;Issue}_{t-1}+\beta_{2}CO_{2}\;{+\;\beta }_{3}GDP\\ \;+\;\beta_{4}Health\;Expenditure+\;\beta_{5}Population+\;\varepsilon \end{array}$$

In this equation, β represents the coefficients of independent variables. ϵ is referred as error terms that are not detected from data. We use PM2.5 to as a proxy of health issues, as it seems one of the major air pollutant particles. Previous studies have missed the case of Saudi Arabia. Nearly, none of the study is about public health issues and prediction for Saudi Arabia. However, it is important to examine the causes and predictions of public health issues in Saudi Arabia. I will help the policymakers to drive an accurate health policy.

### Support Vector Machine

Support vector machine (SVM) has been occurred as supervised classification tool for sorting and regression solutions. In the latest years, this methodology has gained interest and able to end up many suitable solutions. SVM is being considered as robust and accurate technique among ML algorithms. Different studies opt this method with ANN for health-related issues (49–52). According to Byvatov et al. (53), SVM outperformed and outcomes are more robust with least errors. The regression model is produced through a sequence of high-dimensional functions. The formulations between equations (2) and (9) explain SVR technique from a mathematical aspect.


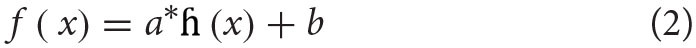


where 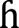
(x) signifies the kernel transformation function for the inputs and *a* and *b* are parameters. The coefficients are designed by lessening the standardized risk function that is given under:


(3) $$\begin{array}{r} R\left(f\right)=c\frac{1}{n\;}\overset{n}{\underset{i=1}{\sum }}L\varepsilon \left(y_{i},f\left(x_{i}\right)\right)+\frac{1}{2}||a^{2}|| \end{array}$$

Where, ε is indicated the tolerance value.


(4) $$L_{e}\left(y,\;f\left(x\right)\right)=\{\begin{array}{c} 0\\ \left|y-f\left(x\right)\right|-\varepsilon \end{array}\;\;\left|\begin{array}{c} y-f\left(x\right)<\varepsilon \\ y-f\left(x\right)\geq \varepsilon \end{array}\right|$$

In the above equation, *L*_ε_(*y, f*(*x*)) is marked a ε insensitive loss function. The loss is considered as zero if the projected value is inside the ε range. Corresponding to the lenient margin hyperplane, we need to present two slack variables, namely, $\emptyset_{i},\emptyset_{i}^{*}$, representing the positive and negative deviances, respectively, out of the ε range. Equation (2) is reformulated in the following restraint formula:


(5) $$\begin{array}{r} Min\;\frac{1}{2}||a|{|}^{2}+C\overset{n}{\underset{i=1}{\sum }}\left(\emptyset_{i}+\emptyset_{i}^{*}\right) \end{array}$$

Subject to:


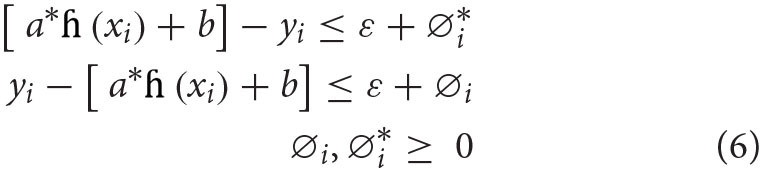


The following form solves this restraint optimization solution:


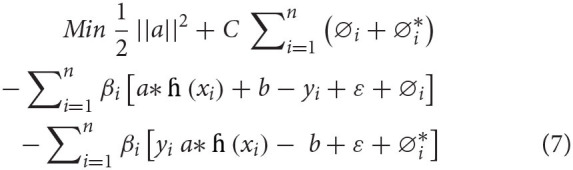


The dual form is mentioned under:


$$\begin{array}{l} \max\overset{n}{\underset{i=1}{\sum }}y_{i}\left(\emptyset_{i}-\emptyset_{i}^{*}\right)-\varepsilon \sum_{i=1}^{n}\left(\emptyset_{i}-\emptyset_{i}^{*}\right)\\ \;-\frac{1}{2}\sum_{i=1}^{n}\sum_{j=1}^{n}y_{i}\left(\emptyset_{i}-\emptyset_{i}^{*}\right)\\ \;\times \left(\emptyset_{j}-\emptyset_{j}^{*}\right)k\left(x_{i}x_{j}\right)subjectto:\\ \;\overset{n}{\underset{i=1}{\sum }}\left(\emptyset_{i}-\emptyset_{i}^{*}\right)=0;\\ 0\leq \emptyset_{i}\leq C;0\leq \emptyset_{i}^{*}\leq C;where\;i=1,2,3\ldots \ldots \ldots n \end{array}$$

Dependable with the Karush–Kuhn–Tucker theorem, our regression equation is stated as:


(8) $$\begin{array}{r} f\left(x\right)=\left(\emptyset_{i}-\emptyset_{i}^{*}\right)k\left(x_{i}x_{j}\right)+b \end{array}$$

*k*(*x*_*i*_*x*_*j*_) represents a kernel function, which equals the value of input of two vectors, namely, *x*_*i*_ *and x*_*j*_, in the feature space *h*(*x*_*i*_) *and h*(*x*_*j*_).

Among some most utilized kernel functions, such as radial basis kernel, polynomial kernel, sigmoidal kernel, and linear kernel, this study used polynomial kernel function to examine the health issues. Moreover, polynomial kernels have been widely utilized in diverse applications (54). The polynomial kernel with an order of *d* and constant of α_1_and α_2_ can be formulated in equation (9) as:


(9) $$\begin{array}{r} k\left(x_{i}x_{j}\right)={\left(\;\alpha_{1}x_{i}x_{j}+\alpha_{2}\right)}^{d} \end{array}$$

Overall SVR method centers around the hyperparameters (*C*, ε) and the kernel parameters (*d*). These parameters and their determining them support correctness of the SVR model. The identification of all the three parameters significantly influences the estimation accuracy of SVR tools. More unambiguously, *C* provides the balance among the training error and the model strength. If *C* takes too huge a value, the observed risk of the objective purpose will be lessened. According to Vapnik's, ε is systemically created minimal radius tube that values of errors, which are lesser than certain threshold, are ignored. Smaller values of ε determine low tolerance error, so it effects the support vector and ultimately the solutions (55).

### Artificial Neural Network Models

Artificial neural network (ANN) is stimulated by the neurological functions caused by human brain and is articulated on the human being intellectual system. Scholars in numerous fields indicated a great attention in ANN as of its aptitude to discover resolutions under multidimensional, non-linear, and complex statistics. ANN can effortlessly manage with non-linear models and under imperfect data arrangement plus deliver fruitful consequences. ANNs comprise a great quantity of computational fundamentals (neurons) networking across subjective associates. ANN displays some features such as the functioning norm of the human brain and later learning multipart knowledge configurations, as it simplifies this information to apply in several circumstances. This method can be classified into supervised and unsupervised model; ANN is better performed in predicting results. ANN is highly flexible and its capability to perform diverse relationship from input to output. This technique outperforms even data set is unclear and missing. ANN is broadly used as a practical substitute (56). The samples under this technique are divided into two sets that named as training data and testing data. Testing data is applied to perform the testing process and training data gives the link between output and input (57). In order to acquire the ideal network architecture, different groupings are evaluated. Among these combinations, a factor is to identify proper transfer function. We have applied a hyperbolic tangent sigmoid transfer function as stated in equation (10):


(10) $$\begin{array}{r} f\left(x\right)=\frac{1-e^{-x}}{1+e^{-x}} \end{array}$$

where *x* is the weighted input summation of the hidden layer and *f*(*x*) is the output of the hidden layer.

The most extensively employed learning algorithm is the backpropagation algorithm (58). The major idea behind using backpropagation algorithm is to reduce the sum of square errors backward propagating through the ANN. The Levenberg–Marquardt algorithm is generally applied to calculate the weights of ANN in backpropagation algorithm. This is derived from Newton's method (59) and for minimizing a function *V*(*d*) with respect to the vector d given in equation (11):


(11) $$\begin{array}{r} ▽\left(d\right)={-\left[\nabla^{2\;}V\left(d\right)\right]}^{2}\nabla v\left(d\right) \end{array}$$

[∇^2^*V*(*d*)] denotes the Hessian matrix and ∇*V*(*d*) is the gradient vector.

**Figure 3:**
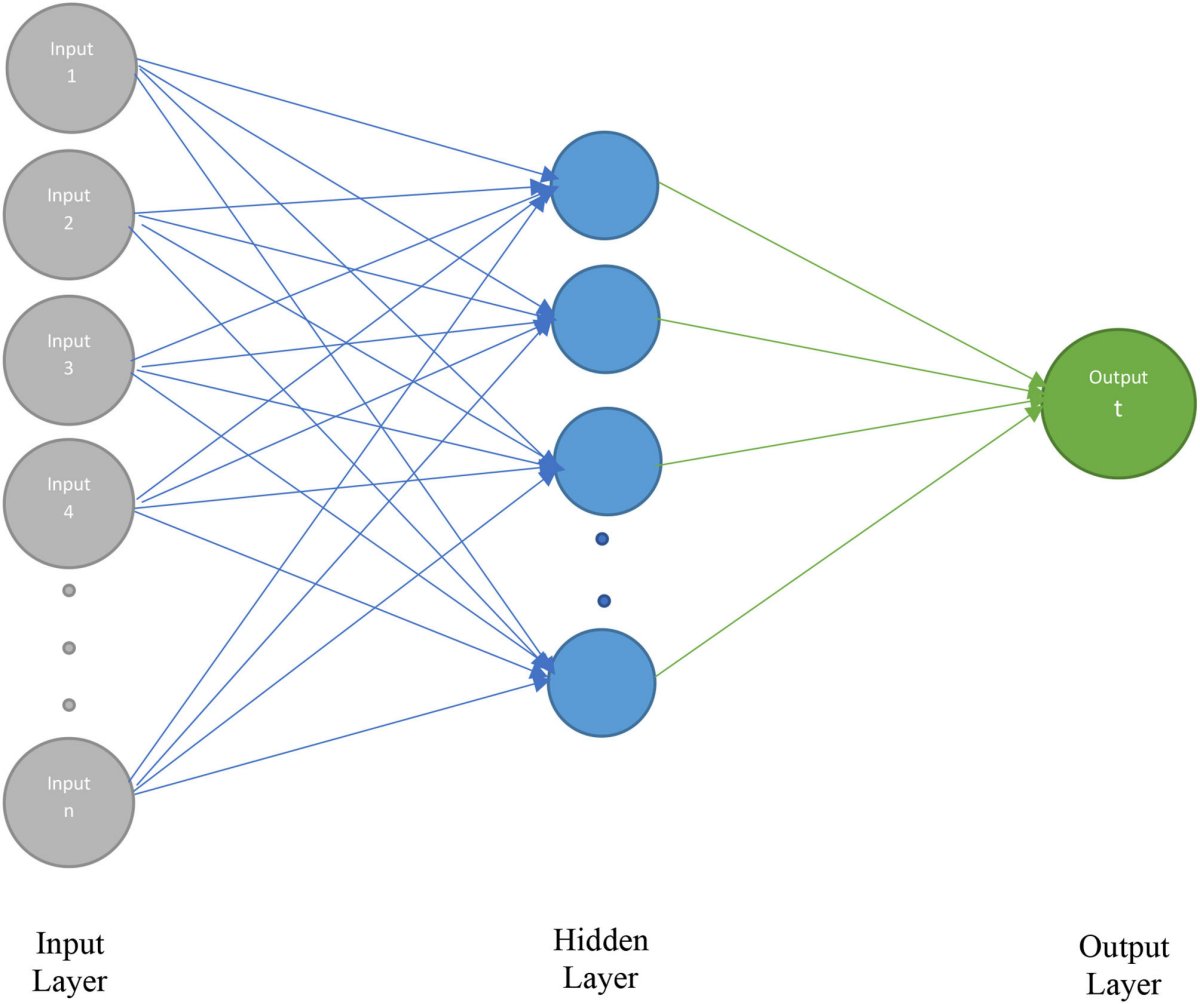
Architecture of artificial neural network (ANN).

Figure 3 elaborates the input, hidden, and output layers of ANN.

### Proposed Method

**Figure 4:**
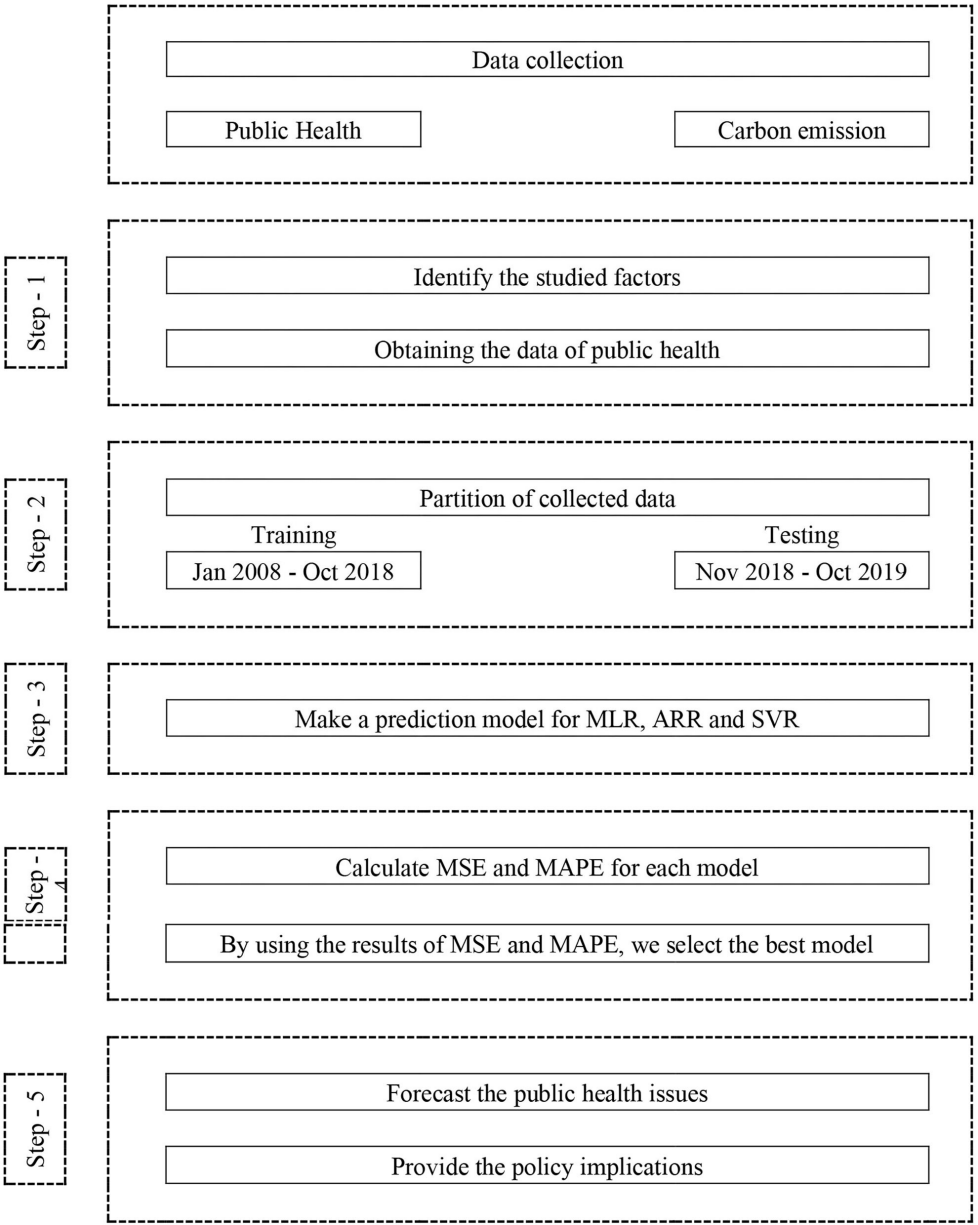
The proposed steps for the methodology of this study.

The anticipated forecasting method is given in Figure 4. This method incorporates three main stages: First, it identifies the highly suitable method for forecasting the health issues in Saudi Arabia. Second, it determines the topmost affecting variable in forecasting the risk in health matters. Third, it refers some solutions to decision-makers and health workers, so it helps to beat the risk issues.

Stage 1: The inputs of the suggested anticipating model are exposure to PM2.5 of past years, *CO*_2_, *GDP*, health expenditures, and selected age for population. The primary phase comprises collection of data, its examination, and abstraction of its structures.

Step 2: The next step contains dividing the data set into the training and testing data sets. These data set will aid in identifying the model's performance in latter stage and enable to determine the model restrictions.

Step 3: In this step, different machine learning tools, which include SVR, ANN, and MLR, are applied to the data set in order to get the predicting model for health issues in Saudi Arabia.

Step 4: After constructing the forecasting model, this step compares the performance of each model attained by different machine learning techniques. The comparison is based on the mean absolute percentage error (MAPE), which assists in adjusting parameters to get more correct forecasting outputs.

Step 5: As parameters are adjusted in previous step, this last stage foresees risks outcomes in health issue for the next years that benefits policymakers and health workers to identify the problems and major variables to be noticed.

## Results and Discussion

As discussed in the previous section, our first step includes an assessment of previous years' forecasted health issues that were aroused by some other variables. Using the data from 2008 to 2019, Figure 5 clearly defines that, according to time plotted, 2010 was an important year as the rise in health exposure to PM2.5 increased each month. The following years had the same pattern. The main contributors to the poor air quality are the carbon output from automobiles and manufacturing plants, as well as the effects of natural uncertain dust storms. In Saudi Arabia, dust storms are worse during spring from March to May every year (60).

**Figure 5:**
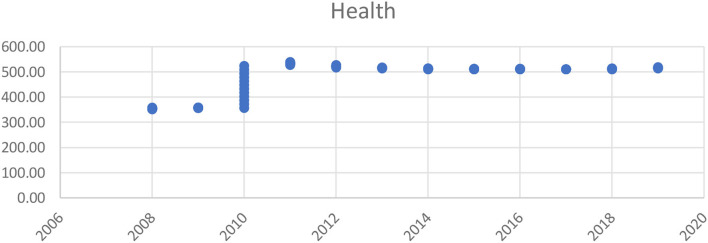
Health issue since 2006–2020.

**Figure 6:**
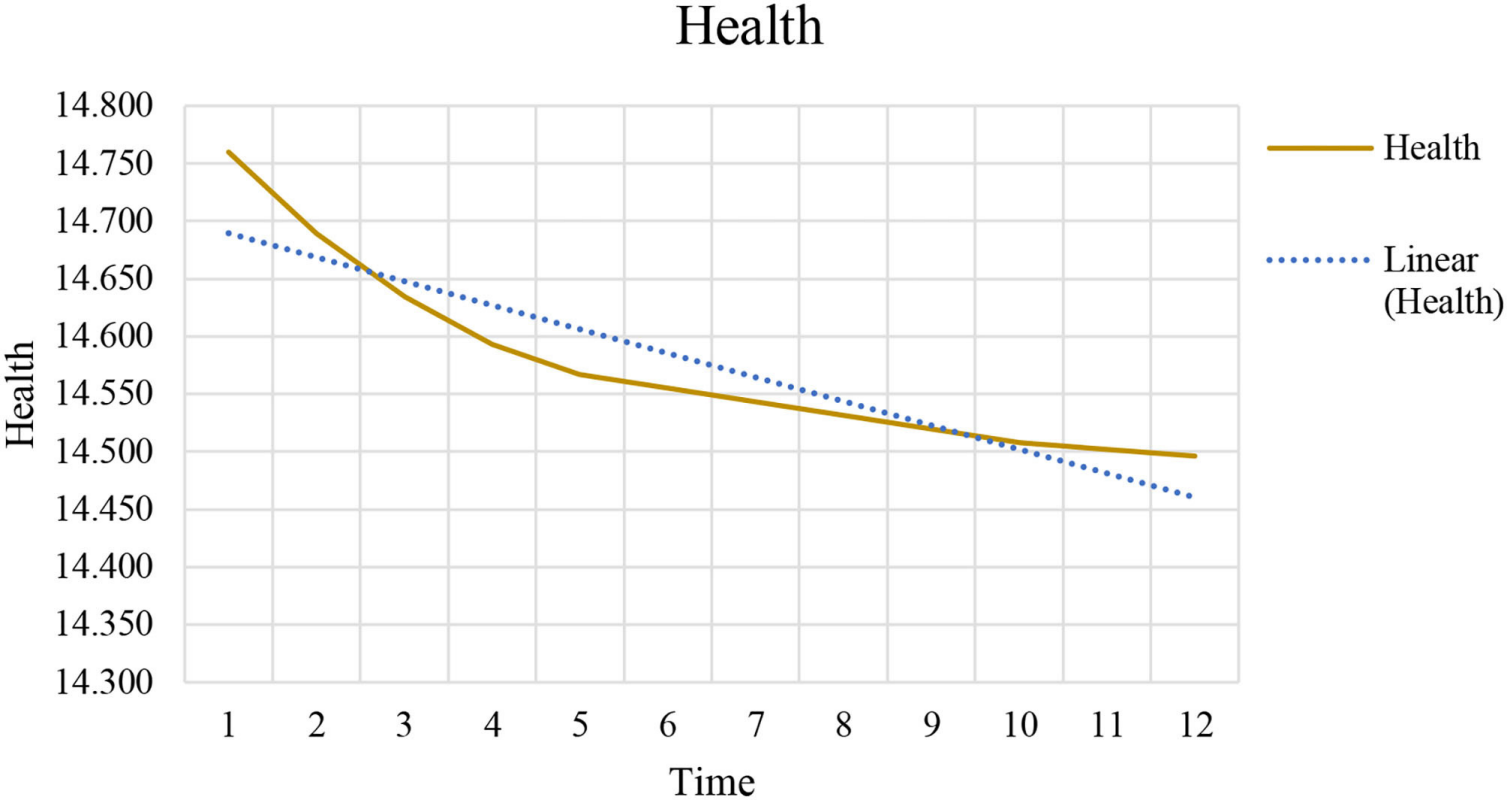
Plot of health issues and prediction of testing data.

Other than historical health data, more variables should be considered to identify and assess health risks. For this purpose, we incorporated variables such as *CO*_2_, *GDP*, *health expenditure*, and *population*. Figure 6 predicts the testing data by linear plotting. Its linear trend helps to forecast a correct and precise model.

Moreover, we applied tests to analyze the autocorrelation values from lag 1 to lag 15, as shown in Table 3. The results depicted that lag 11 had the highest autocorrelation value of 0.92. Forecasting the time series health issues suggested that inputs include *CO*_2_, *GDP*, *health expenditure*, and lag11 (lag health) in the proposed model. Upon further analysis, we performed a stepwise regression model to identify the best predicting variables that affect health in Saudi Arabia.

**Table 3:** Autocorrelation of health issues [exposure to particulate matter 2.5 (PM2.5)].

**Lags**	**1**	**2**	**3**	**4**	**5**	**6**	**7**	**8**	**9**	**10**	**11**	**12**	**13**	**14**	**15**
Correlation	0.03	0.16	0.45	0.02	−0.16	−0.27	−0.23	−0.41	−0.05	0.47	0.92	0.43	0.12	−0.54	−0.26

*The value of autocorrelation is higher at lag 12, however, we use lag 12 as independent variable along with other independent variables*.

### Splitting the Data Set

The anticipated procedure to forecast health risks requires other variables as mentioned by regression model. Each variable is important to predict the risk of health issues. Continuing the procedure, the data set is divided into the training and testing data sets for the period of 1/1/2008 to 31/12/2018 for the proposed model.

Table 4 shows the unit root test results applied on our data set. Prior to application of MLR technique, we analyzed the existence of stationary data set. We applied Augmented Dickey–Fuller (ADF) and Kwiatkowski–Phillips–Schmidt–Shin (KPSS) tests and our results confirmed that all the variables are stationary at level. Hence, we can apply our MLR technique following our selected machine learning tools such as ANN and SVR. Demonstrating stepwise regression results in Table 5, we found that health expenditures, *CO*_2_, and *GDP* are significantly contributive toward the health issues forecasting. Since *population* is considered to be insignificant in its role of prediction, Saudi Arabia faces challenges of carbon emission and its impact on public health because of its oil production (39). As one of the richest countries in the world, Saudi Arabia is investing huge funds into their public sectors such as education and health. In such scenario, the government provides health opportunities by increasing the health benefits at door steps (61). The effect of *GDP* with its oil income is higher, directly reducing the investment in health expenditures, so it is an important factor in recognizing the health risk in upcoming years. The population is rising, which increases the health expenditure (33); but, in case of Saudi Arabia, it is not significant predictor for health risk. *Health expenditure* of the country is negatively related to health issues, which highlight that more health expenditure leads to less public health risks. Increased health spending will decrease health risks in Saudi Arabia for the next few years. We also found that *CO*_2_ has a positive effect on health since hazard emissions in the environment affect exposure to PM2.5, raising the pollution level and creating health and environmental issues. Considering the effect of *GDP*, it has a negative relationship with health because of progress in other sectors that result in less spending toward health departments.

**Table 4:** Unit root.

**Variable**	**ADF (*p*)**	**Result**	**KPSS (*p*)**	**Result**
Health	0.001	Stationary	0.742	Stationary
*Health Expenditure*	0.000	Stationary	0.993	Stationary
*CO*_2_	0.027	Stationary	0.254	Stationary
*GDP*	0.000	Stationary	0.684	Stationary
*Population*	0.000	Stationary	0.218	Stationary

*The null hypothesis of ADF shows the presence of unit root in series. For KPSS, the null hypothesis presents the series are stationary. We found all the variables are stationary at level*.

**Table 5:** Stepwise regression results.

**Variable**	**Coefficient**	**Prob**.
*Healthissues*_*t*−1_	0.992***	0.000
*Health Expenditure*	−0.050**	0.019
*CO*_2_	0.075**	0.011
*GDP*	−0.081***	0.000
*Population*	−0.093	0.742
Constant	3.686***	0.000
R-squared		0.997
Adjusted R-squared		0.997
S.E. of regression		0.008
Sum squared residual		0.008
Log likelihood		446.950
F-statistic		8455.636
Prob(F-statistic)		0.000

The *** and ** symbols indicates the level of significance at 1 and 5 respectively.

**Figure 7:**
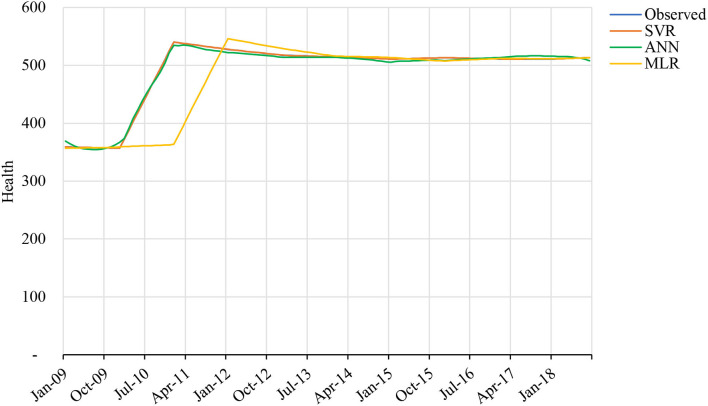
The graph shows the real health issues and forecasts from multiple linear regression (MLR), ANN, and support vector regression (SVR) for training data.

Figure 7 is the graphical representation of our results that prove that SVR forecast on the data set is more accurate, whereas MLR and ANN are lacking behind as in prediction model. Table 6 demonstrates that SVR is the most precise forecasting model of mean squared error (MSE) with value 0.008 and MAPE with 0.014 for training data. Similarly, testing data also showed the lowest value of MSE with 0.010 and MAPE with 0.015 compared to MLR and ANN. In this study, ANN is with highest MAPE of 0.725 in predicting the future health issues. The proposed SVR model with a polynomial cubic kernel function can be implemented as a significant policymaking tool in forecasting the upcoming risk in health issues and what steps to take.

**Table 6:** Mean squared error (MSE) and mean absolute percentage error (MAPE) statistics to evaluate the forecasting performance.

	**ANN**		**SVR**		**MLR**	
	**MSE**	**MAPE**	**MSE**	**MAPE**	**MSE**	**MAPE**
Training data	15.309	0.725	0.008	0.014	5.847	0.605
Testing data	0.233	4.838	0.010	0.015	0.186	0.163

**Figure 8:**
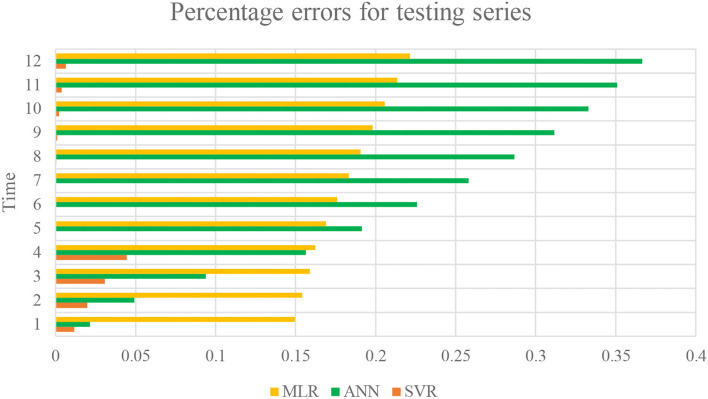
The percentage errors of MLR, ANN, and SVR models for testing data.

**Figure 9:**
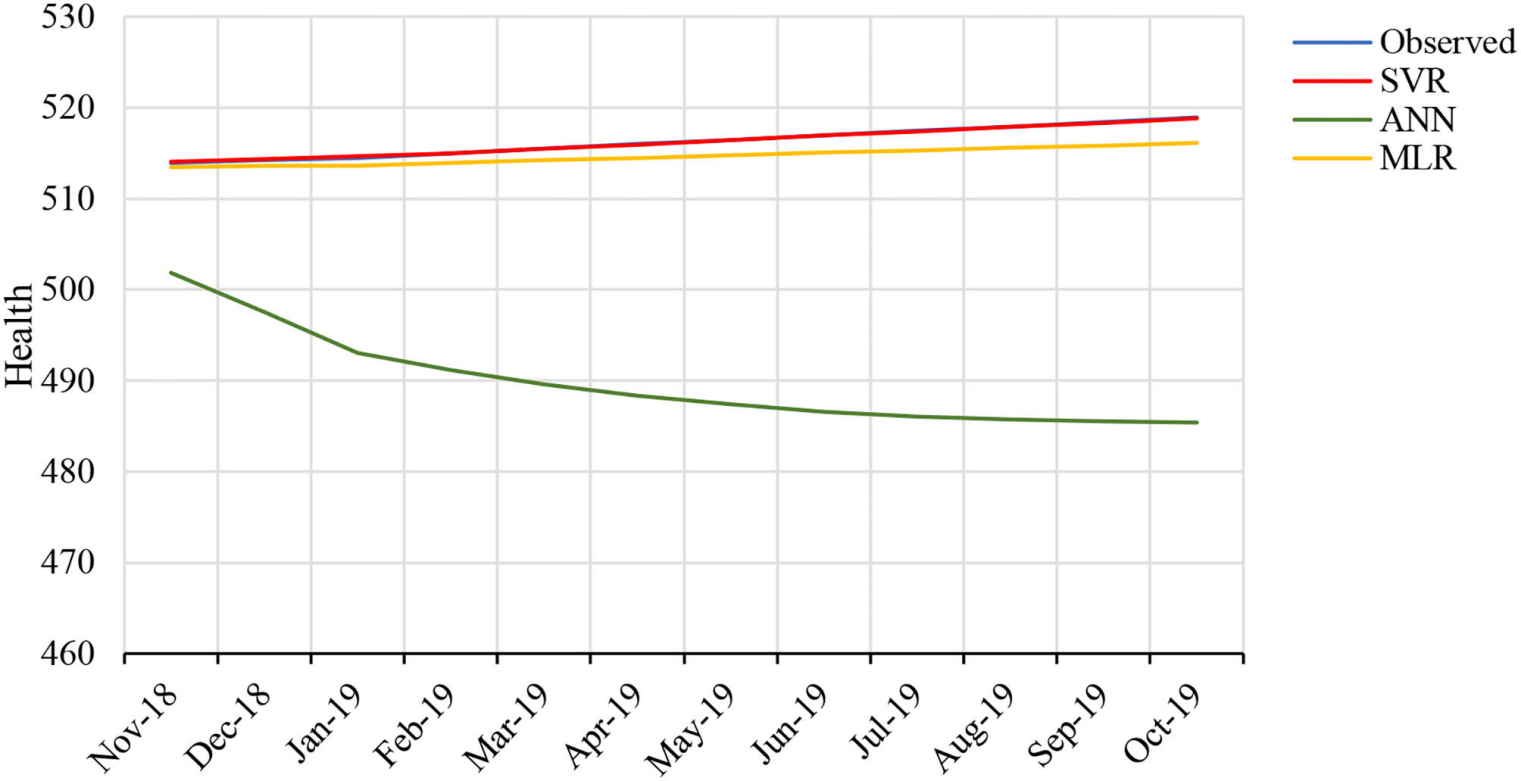
The graph shows the real health issues and forecasts from MLR, ANN, and SVR for testing data.

Figure 8 determines that prediction of health risks has lower percentage in SVR techniques than in ANN and MLR. ANN has highest percentage of errors. Figure 9 represents the forecast testing data derived from SVR and this graph depicts that the data are the closest to observed data. Accuracy is more justified to be used as predicting model for health issues in this study.

**Figure 10:**
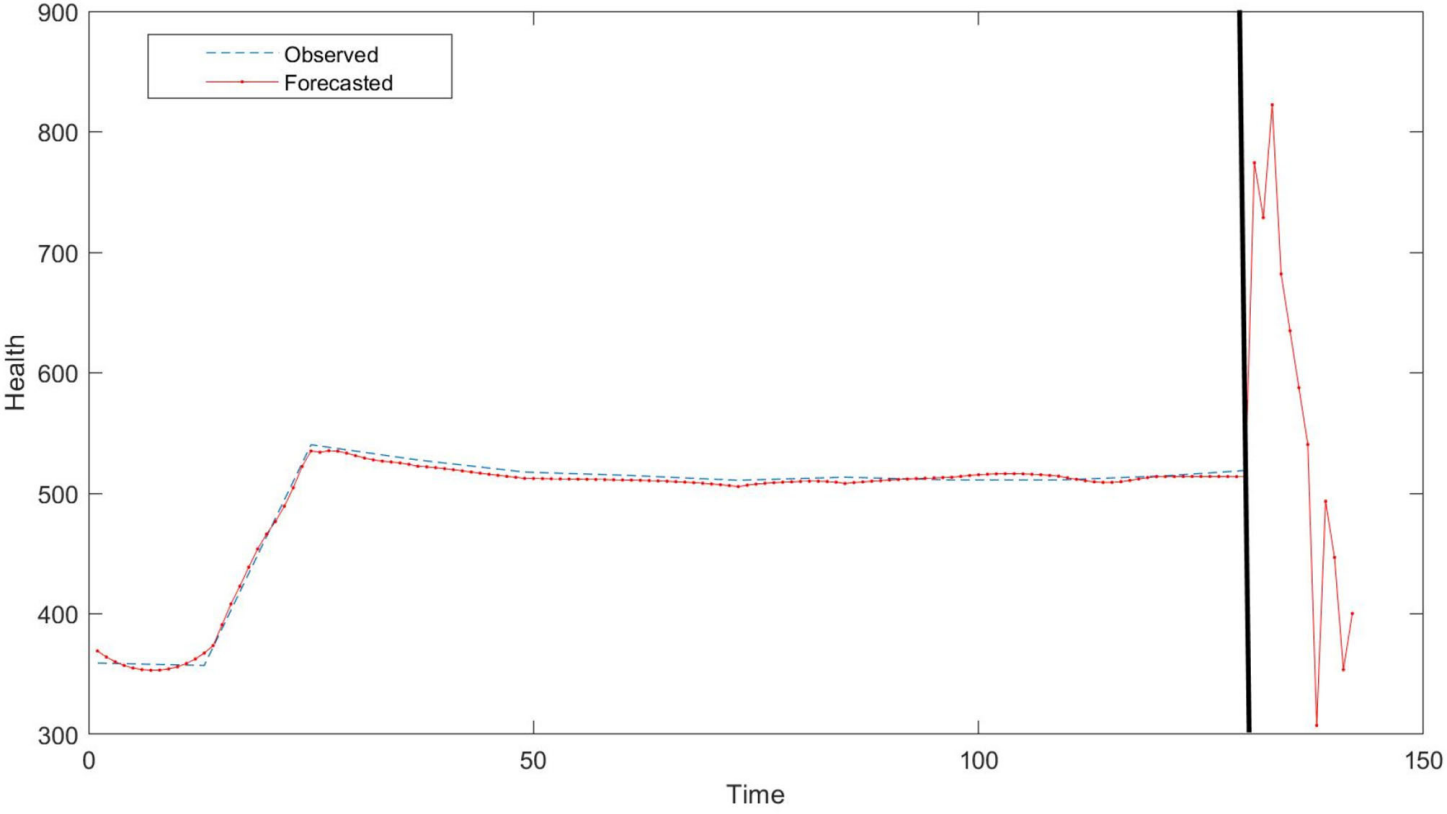
Forecasted health issues from October 2018 to October 2019.

Since our results support the SVR model for prediction, we use the SVR machine learning tool to forecast the health issues in coming months. Figure 10 depicts that health issues will rise in the upcoming months, but health issues will decrease, compensating for the rise. We attribute this to the government's precaution and increased spending in health-related policies.

## Conclusion and Implications

The rise in health issues resulting from environmental factors stimulates the concentration of researchers, since this is an important policy or financing department for countries. This study incorporates forecasting of health issues of KSA and highlights the major impacts on public health. The people of KSA are also facing rising health issues due to their personal lifestyle and poor eating habits. Another factor is hazardous emissions that cause air pollutants, which are vital to understanding the health risks. In this study, we used four variables, which are proposed forecasting factors for health-associated risks in Saudi Arabia. These factors include carbon emissions, GDP, population, and health expenditures. This study applied multilinear regression to understand and find the primary concern of forecasted health issues. Our statistical results show a strong negative relationship between health issues, GDP, and health expenses of the country. This demonstrates that GDP and health financing of the country can decrease health issues. We believe that, as a participant of the National Transformation Program 2020, the Ministry of Health declared advanced funds, improvements, and development of the Saudi Arabian healthcare plan. Moreover, investments in the health department started in 2010 and to date are considered to be an important part of policymaking and to finance departments. According to Tyrovolas et al. (62), KSA mortality rates decrease more during the period of 2010 to 2017 than from 1990 to 2010. Our analysis found that carbon emissions negatively affect health risks, since PM2.5 arises from hazardous emissions, which contribute to major health issues. Air pollution is highly affiliated with cardiovascular illnesses, strokes, respiratory diseases, and neurological development in children (2–4). Since population is found to be insignificant and shows no association with health issues, KSAs rising population might increase health expenses because of free medical services, but healthcare investments are enough to fix this risk.

In this study, another important finding includes the accurate forecasting model for health issues risk in KSA. We applied MLR, SVM, and ANN for this purpose, which resulted in SVM to be more accurate than ANN and MLR techniques. As mentioned in Table 6, SVM has the most accurate results with MSE of 0.008 and 0.010 for training and testing data as compared to ANN and MLR. On this basis of accuracy selection of SVM model, we forecasted that medical issues will rise in the beginning of next year. Drastic reduction has also been forecasted by the year October 2019, giving a view of the National Transformation Program 2020 activities effect on this reduction.

As a result of this study's findings, we recommend that policymakers concentrate on variables such as carbon emission, GDP, and health expenses in their decision-making. These are highly contributive factors for health issues risks and cannot be ignored to combat this concern. Financing for health expenses needs investments, hence enhancing GDP is a vital factor to increased government spending. Decision-makers can use the machine learning tool SVM to forecast upcoming years' health issues before determining policy or financing decisions, so they can achieve more accurate results and less error performance. This study can be further enhanced by comparing the other GCC countries and determining the most impactful factor in decision-making. In another way, this study has missing pandemic time period and can incorporate pre- and post-coronavirus disease 2019 (COVID-19) situation of the country. This study can further be improved by introducing variables such as dividing population into gender, age below 35 or above 35 years, and children. This could highlight whether population is still an insignificant factor in forecasting health issues or if some classification is not contributing significantly toward health risk issues. Importantly, further studies can use other proxies of health issues such as exposure to carbon.

## Data Availability Statement

Publicly available datasets were analyzed in this study. This data can be found here: WDI and OECD.

## Author Contributions

HA-A formulates the idea, write the manuscript, and analyse the results and review the manuscript.

## Funding

This work was funded by the University of Jeddah, Jeddah, Saudi Arabia, under grant No. (UJ-22-DR-24).

## Conflict of Interest

The author declares that the research was conducted in the absence of any commercial or financial relationships that could be construed as a potential conflict of interest.

## Publisher's Note

All claims expressed in this article are solely those of the authors and do not necessarily represent those of their affiliated organizations, or those of the publisher, the editors and the reviewers. Any product that may be evaluated in this article, or claim that may be made by its manufacturer, is not guaranteed or endorsed by the publisher.

## References

[1] Lustig N Gonzalez E. Investigating in Health for Economic Development. In: Commission on Microeconomics and Health Macroeconomics and Health (2004).

[2] Shah AS Lee KK McAllister DA Hunter A Nair H Whiteley W. Short term exposure to air pollution and stroke: systematic review and meta-analysis. BMJ. (2016) 354:i4851. 10.1136/bmj.i4851 25810496 PMC4373601

[3] Atkinson RW Kang S Anderson HR Mills IC Walton HA. Epidemiological time series studies of PM2.5 and daily mortality and hospital admissions: a systematic review and meta-analysis. Thorax. (2014) 69:660–5. 10.1136/thoraxjnl-2013-204492 24706041 PMC4078677

[4] Suades-González E Gascon M Guxens M Sunyer J. Air pollution and neuropsychological development: a review of the latest evidence. Endocrinology. (2015) 156:3473–82. 10.1210/en.2015-1403 26241071 PMC4588818

[5] Broome RA Fann N Cristina TJN Fulcher C Duc H Morgan GG. The health benefits of reducing air pollution in Sydney, Australia. Environ Res. (2015) 143:19–25. 10.1016/j.envres.2015.09.007 26414085

[6] Young Y Alharthy A Hosler AS. Transformation of Saudi Arabia's health system and its impact on population health: what can the USA learn? Saudi J Health Syst Res. (2021) 1:93–102. 10.1159/000517488

[7] The World Bank DataBank - World Development. Indicators (2013). 10.1596/978-0-8213-9616-2

[8] Al-Aly Z Bowe B. The road ahead for research on air pollution and kidney disease. J Am Soc Nephrol. (2021) 32:260–2. 10.1681/ASN.2020121713 33462082 PMC8054881

[9] Kan H Chen B Zhao N London S Song G Chen G. Part 1. A time-series study of ambient air pollution and daily mortality in Shanghai, China. Res Rep Health Eff Inst. (2010) 154:17–78. 21446211

[10] Carré J Gatimel N Moreau J Parinaud J Léandri R. Does air pollution play a role in infertility?: a systematic review. Environ Health. (2017) 16:82. 10.1186/s12940-017-0291-8 28754128 PMC5534122

[11] Juginović A Vuković M Aranza I Biloš V. Health impacts of air pollution exposure from 1990 to 2019 in 43 European countries. Sci Rep. (2021) 11:22516. 10.1038/s41598-021-01802-5 34795349 PMC8602675

[12] Bandara A. Emerging health issues in Asia and the Pacific: implications for public health policy. Asia Pac Dev J. (2006) 12:33–58. 10.18356/9dbe1347-en

[13] Thomson P Jaque S. Understanding Creativity in the Performing Arts, in Creativity and the Performing Artist, 2017. Academic Press Inc. (2016). 10.1016/B978-0-12-804051-5.00001-9

[14] Todd A Thomson K Hillier-Brown F McNamara C Huijits T Bambra C. The effects of public health policies on health inequalities in European welfare states. Eur J Public Health. (2017) 27:ckx187.683. 10.1093/eurpub/ckx187.68327059307

[15] Brumberg HL Karr CJ. Ambient air pollution: health hazards to children. Pediatrics. (2021) 147:e2021051484. 10.1542/peds.2021-051484 34001642

[16] Buka I Koranteng S Osornio-Vargas AR. The effects of air pollution on the health of children. Paediatr Child Health. (2006) 11:513–6. 10.1093/pch/11.8.513 19030320 PMC2528642

[17] Shi L Feng X Qi L Xu Y Zhai S. Modeling and predicting the influence of PM 2.5 on children's respiratory diseases. Int J Bifurcat Chaos. (2020) 30:2050235. 10.1142/S0218127420502351

[18] Gilliland F Avol E McConnell R Berhane K Gauderman WJ Lurmann FW. The effects of policy-driven air quality improvements on children's respiratory health. Res Rep Health Eff Inst. (2017) 190:1–75. 31898879 PMC7266378

[19] Ghorani-Azam A Riahi-Zanjani B Balali-Mood M. Effects of air pollution on human health and practical measures for prevention in Iran. J Res Med Sci. (2016) 21:65. 10.4103/1735-1995.189646 27904610 PMC5122104

[20] Manisalidis I Stavropoulou E Stavropoulos A Bezirtzoglou E. Environmental and health impacts of air pollution: a review. Front Public Health. (2020) 8:14. 10.3389/fpubh.2020.00014 32154200 PMC7044178

[21] Schwela D. Air pollution and health in urban areas. Rev Environ Health. (2000) 15:13–42. 10.1515/REVEH.2000.15.1-2.13 10939084

[22] Qian Z He Q Lin HM Kong L Zhou D Liang S. Part 2. Association of daily mortality with ambient air pollution, and effect modification by extremely high temperature in Wuhan, China. Res Rep. (2010) 154:91–217. 21446212

[23] World Health Organization (2016). Household Air Pollution and Health. WHO Media Centre. Available online at: https://www.who.int/data/gho/data/themes/topics/topic-details/GHO/household-air-pollution#:~:text=Household air pollution is generated,environmental contributors to ill health.

[24] Tsakas. PM, Siskos PA, Siskos AP. Indoor Air Pollutants and the Impact on Human Health. In Chemistry, Emission Control, Radioactive Pollution and Indoor Air Quality (2011).

[25] Eisner MD Balmes JR. Murray and nadel's textbook of respiratory medicine. In: Murray and Nadel's Textbook of Respiratory Medicine. Elsevier. (2010). p. 1601–1618. 10.1016/B978-1-4160-4710-0.00067-5

[26] Sarwar S Streimikiene D Waheed R Mighri Z. Revisiting the empirical relationship among the main targets of sustainable development: growth, education, health and carbon emissions. Sustain Dev. (2021) 29:419–40. 10.1002/sd.2156

[27] Sarwar S Alsaggaf MI Tingqiu C. Nexus among economic growth, education, health, and environment : dynamic analysis of world-level data. Front Public Health. (2019) 7:307. 10.3389/fpubh.2019.00307 31709219 PMC6823185

[28] AlQuaiz AM Siddiqui AR Qureshi RH Fouda MA AlMuneef MA Habib FA. Women Health in Saudi Arabia: A review of non-communicable diseases and their risk factors. Pak J Med Sci. (2014) 30:422–31. 10.12669/pjms.302.4378 24772156 PMC3999023

[29] Subhan MM Al-Khlaiwi T Ghandourah SO. Smoking among health science university students in Riyadh, Saudi Arabia. Saudi Med J. (2009) 30:1610–2. 10.1007/s10900-014-9909-8 19936431

[30] Nafees AA Taj T Kadir MM Fatmi Z Lee K Sathiakumar N. Indoor air pollution (PM2.5) due to secondhand smoke in selected hospitality and entertainment venues of Karachi, Pakistan. Tob Control. (2012) 21:460–4. 10.1136/tc.2011.043190 21680561

[31] Al Daajani MM Al-habib DM Ibrahim MH Al Shewear NA Fagihi YM Alzaher AA. Prevalence of health problems targeted by the national school-based screening program among primary school students in saudi arabia, 2019. Healthcare. (2021) 9:1310. 10.3390/healthcare9101310 34682990 PMC8544408

[32] Moradi-Lakeh M El Bcheraoui C Tuffaha M Daoud F Al Saeedi M Basulaiman M. The health of Saudi youths: current challenges and future opportunities. BMC Fam Pract. (2016) 17:26. 10.1186/s12875-016-0425-z 26946327 PMC4779578

[33] Al-Hanawi MK Khan SA Al-Borie HM. Healthcare human resource development in Saudi Arabia: emerging challenges and opportunities—a critical review. Public Health Rev. (2019) 40:1. 10.1186/s40985-019-0112-4 30858991 PMC6391748

[34] Alkadi S. The healthcare system in Saudi Arabia and its challenges: the case of diabetes care pathway. J Health Inform Dev Ctries. (2016) 10:1–29. 10.2147/NDT.S48782 23966783 PMC3743653

[35] Syed Meraj A Mohammed Al M. A study on the prevalence of risk factors for diabetes and hypertension among school children in Majmaah, Kingdom of Saudi Arabia. J Public Health Res. (2017) 6:829. 10.4081/jphr.2017.829 29071251 PMC5641670

[36] Walston SL Al-Harbi Y Al-Omar B. The changing face of healthcare in Saudi Arabia. Ann Saudi Med. (2008) 28:243–50. 10.5144/0256-4947.2008.243 18596400 PMC6074349

[37] Donohoe M. Causes and health consequences of environmental degradation and social injustice. Soc Sci Med. (2003) 56:573–87. 10.1016/S0277-9536(02)00055-2 12570975

[38] Pimentel D Cooperstein S Randell H Filiberto D Sorrentino S Kaye B. Ecology of increasing diseases: population growth and environmental degradation. Hum Ecol. (2007) 35:653–68. 10.1007/s10745-007-9128-3 32214603 PMC7087838

[39] Mahmood H Alkhateeb TTY Furqan M. Oil sector and CO2 emissions in Saudi Arabia: asymmetry analysis. Palgrave Commun. (2020) 6:88. 10.1057/s41599-020-0470-z

[40] Waheed R Sarwar S Dignah A. The role of non-oil exports, tourism and renewable energy to achieve sustainable economic growth: what we learn from the experience of Saudi Arabia. Struct Change Econ Dyn. (2020) 55:49–58. 10.1016/j.strueco.2020.06.005

[41] Briggs D. Environmental pollution and the global burden of disease. Br Med Bull. (2003) 68:1–24. 10.1093/bmb/ldg019 14757707

[42] Taghvaee SM Omaraee B Taghvaee VM. Maritime transportation, environmental pollution, and economic growth in Iran: Using dynamic log linear model and granger causality approach. Iran Econ Rev. (2017) 21:185–210. 10.22059/IER.2017.62100

[43] Vijayarani S Dhayanand S. Kidney disease prediction using SVM and ANN algorithms. Int J Comput Bus Res. (2015) 6:2229–6166.

[44] Esmaeily H Tayefi M Ghayour-Mobarhan M Amirabadizadeh A. Comparing three data mining algorithms for identifying the associated risk factors of type 2 diabetes. Iran Biomed J. (2015) 22:303–11. 10.29252/ibj.22.5.303 29374085 PMC6058191

[45] Madhuravani B Degala DP Anjaneyulu M Dhanalaxmi B. Prediction exploration for coronary heart disease aid of machine learning. Turk J Comput Math Educ. (2021) 12:312–6. 10.17762/turcomat.v12i9.3042

[46] Hooda S Mann S. Examining the effectiveness of machine learning algorithms as classifiers for predicting disease severity in data warehouse environments. Rev Argent Clin Psicol. (2020) 29:233–51. 10.24205/03276716.2020.824

[47] Son YJ Kim HG Kim EH Choi S Lee SK. Application of support vector machine for prediction of medication adherence in heart failure patients. Healthc Inform Res. (2010) 16:253–59. 10.4258/hir.2010.16.4.253 21818444 PMC3092139

[48] Mello-Román JD Mello-Román JC Gómez-Guerrero S García-Torres M. Predictive models for the medical diagnosis of dengue: a case study in Paraguay. Comput Math Methods Med. (2019) 2019:7307803. 10.1155/2019/7307803 31485259 PMC6702853

[49] Almansour NA Syed HF Khayat NR Altheeb RK Juri RE Alhiyafi J. Neural network and support vector machine for the prediction of chronic kidney disease: a comparative study. Comput Biol Med. (2019) 109:101–11. 10.1016/j.compbiomed.2019.04.017 31054385

[50] Ozkan IA Koklu M Sert IU. Diagnosis of urinary tract infection based on artificial intelligence methods. Comput Methods Programs Biomed. (2018) 166:51–9. 10.1016/j.cmpb.2018.10.007 30415718

[51] Ramli MAM Twaha S Al-Turki YA. Investigating the performance of support vector machine and artificial neural networks in predicting solar radiation on a tilted surface: Saudi Arabia case study. Energy Conversion Manag. (2015) 105:442–52. 10.1016/j.enconman.2015.07.083

[52] Ullah R Khan S Ali H Chaudhary II Bilal M Ahmad I. A comparative study of machine learning classifiers for risk prediction of asthma disease. Photodiagnosis Photodyn Ther. (2019) 28:292–6. 10.1016/j.pdpdt.2019.10.011 31614223

[53] Byvatov E Fechner U Sadowski J Schneider G. Comparison of support vector machine and artificial neural network systems for drug/nondrug classification. J Chem Infm Comput Sci. (2003) 43:1882–9. 10.1021/CI0341161 14632437

[54] Ikeda K. Geometry and learning curves of kernel methods with polynomial kernels. Syst Comput Japan. (2004) 35:41–8. 10.1002/SCJ.10629

[55] Zhang F O'Donnell LJ. Support vector regression. In: Mechelli A, Vieira S, editors. Machine Learning: Methods and Applications to Brain Disorders. London: Academic Press (2019). p. 123–40. 10.1016/B978-0-12-815739-8.00007-9

[56] Tadeusiewicz R. Neural networks: a comprehensive foundation. Control Eng Pract. (1995) 3:746–7. 10.1016/0967-0661(95)90080-2

[57] Hornik K. Some new results on neural network approximation. Neural Netw. (1993) 6:1069–72. 10.1016/S0893-6080(09)80018-X

[58] Wang AJ Ramsay B. A neural network based estimator for electricity spot-pricing with particular reference to weekend and public holidays. Neurocomputing. (1998) 23:47–57. 10.1016/S0925-2312(98)00079-4

[59] Hagan MT Menhaj MB. Brief papers. Brain Cogn. (1996) 32:273–344. 10.1006/brcg.1996.0066

[60] Air Quality in Buraydah Saudi Arabia. International Journal of Innovative Technology and Exploring Engineering (2020).35611745

[61] Farouk AEE Banjar FM Karar HMO Elamin FO. Determinants of public healthcare expenditure in Saudi Arabia. Eur J Pharm Med Res. (2016) 3:85–93.

[62] Tyrovolas S El Bcheraoui C Alghnam SA Alhabib KF Almadi MAH Al-Raddadi RM. The burden of disease in Saudi Arabia 1990–2017: results from the global burden of disease study 2017. Lancet Planet Health. (2020) 4:e195–e208. 10.1016/S2542-5196(20)30075-9 32442495 PMC7653403

